# Physical activity, cardiorespiratory fitness and risk of cutaneous malignant melanoma: Systematic review and meta-analysis

**DOI:** 10.1371/journal.pone.0206087

**Published:** 2018-10-31

**Authors:** Gundula Behrens, Tobias Niedermaier, Mark Berneburg, Daniela Schmid, Michael F. Leitzmann

**Affiliations:** 1 Department of Epidemiology and Preventive Medicine, University of Regensburg, Regensburg, Germany; 2 Department of Dermatology, University Hospital Regensburg, Regensburg, Germany; University of Turin, ITALY

## Abstract

**Background:**

Numerous epidemiologic studies have examined the relation of physical activity or cardiorespiratory fitness to risk of cutaneous melanoma but the available evidence has not yet been quantified in a systematic review and meta-analysis.

**Methods:**

Following the preferred reporting items for systematic reviews and meta-analyses (PRISMA), we identified 3 cohort studies (N = 12,605 cases) and 5 case-control studies (N = 1,295 cases) of physical activity and melanoma incidence, and one cohort study (N = 49 cases) of cardiorespiratory fitness and melanoma risk.

**Results:**

Cohort studies revealed a statistically significant positive association between high versus low physical activity and melanoma risk (RR = 1.27, 95% CI = 1.16–1.40). In contrast, case-control studies yielded a statistically non-significant inverse risk estimate for physical activity and melanoma (RR = 0.85, 95% CI = 0.63–1.14; P-difference = 0.02). The only available cohort study of cardiorespiratory fitness and melanoma risk reported a positive but statistically not significant association between the two (RR = 2.19, 95% CI = 0.99–4.96). Potential confounding by ultraviolet (UV) radiation-related risk factors was a major concern in cohort but not case-control studies.

**Conclusions:**

It appears plausible that the positive relation of physical activity and cardiorespiratory fitness to melanoma observed in cohort studies is due to residual confounding by UV radiation-related risk factors.

**Impact:**

Future prospective studies need to examine the association between physical activity, cardiorespiratory fitness and melanoma after detailed adjustment for UV radiation-related skin damage.

## Introduction

Worldwide, marked annual increases in incidence rates of invasive cutaneous melanoma have been observed across several decades, in particular among populations with low skin pigmentation [1–4]. The most likely reason for the positive long-term trend in melanoma incidence is increased UV (ultraviolet) radiation-related skin damage caused by prolonged sun exposure [5].

UV radiation-related DNA damage is the most relevant modifiable melanoma risk factor, and oxidative stress, chronic inflammation, and impaired immune function all represent contributing factors [6–9]. In contrast, surprisingly little is known about the independent effects of physical activity and cardiorespiratory fitness on melanoma prevention, although physical exercise has a positive impact on numerous biological pathways, such as increased DNA repair capacity, decreased levels of oxidative stress, reduced inflammation, and enhanced immune function [10–13].

Available evidence from epidemiologic studies of physical activity, cardiorespiratory fitness and melanoma risk has been inconsistent, which may be due to differences in study design and incomplete adjustment for potential confounding variables. We therefore conducted a systematic review and meta-analysis of physical activity, cardiorespiratory fitness and melanoma with careful consideration of the influence of study design and adjustment for potential confounding factors, including UV radiation-related skin damage, sun exposure, and sun sensitivity.

## Methods

### Literature search

Following the preferred reporting items for systematic reviews and meta-analyses (PRISMA) [14], two researchers (GB, TN) conducted independent systematic literature searches in PubMed and Web of Knowledge to identify English language epidemiologic studies which assessed physical activity or cardiorespiratory fitness on an individual level and examined the relations of physical activity or cardiorespiratory fitness to newly incident cutaneous melanoma (S1 and S2 Files).

**Fig 1:**
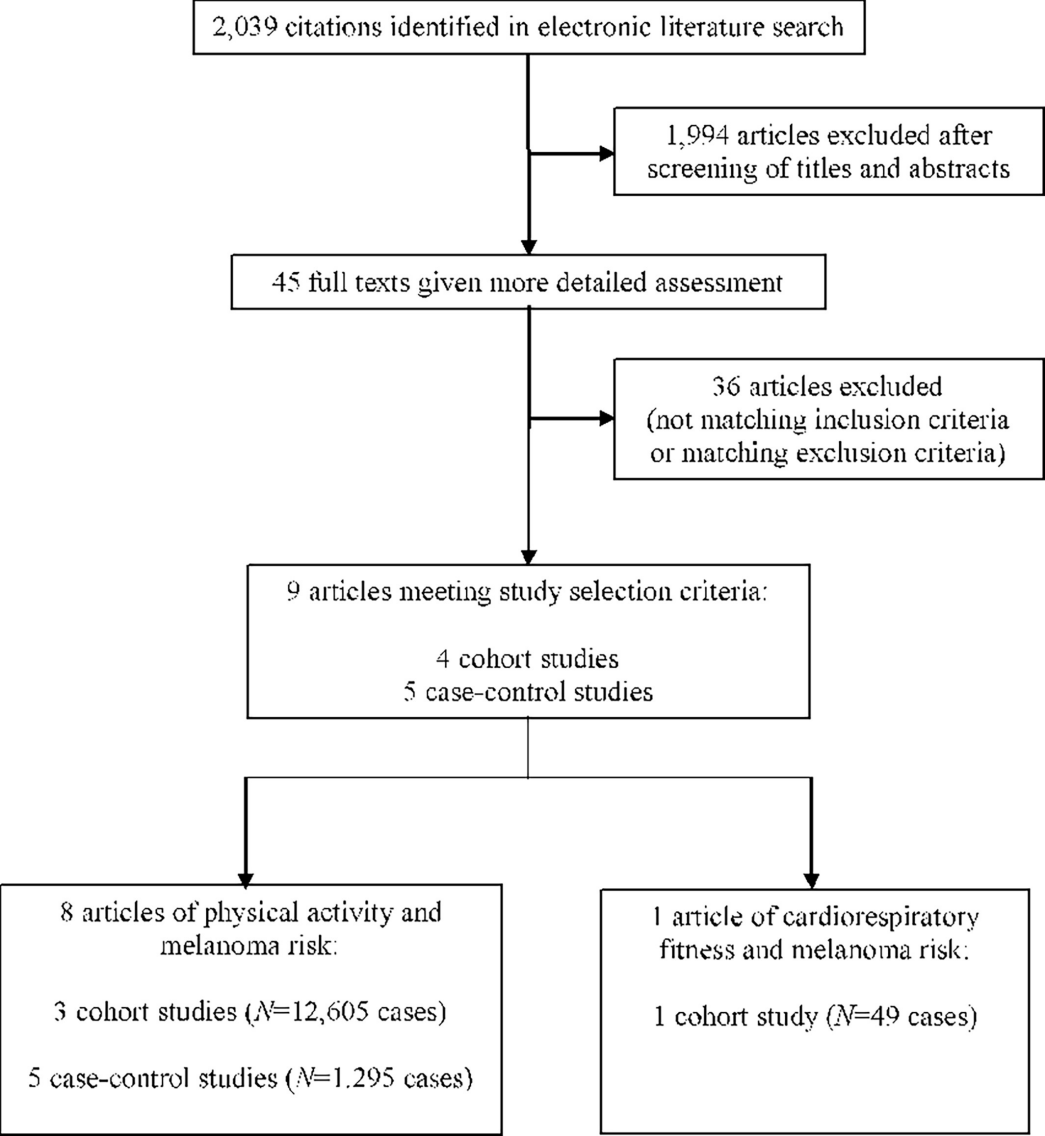
Flowchart of literature search results, last conducted on March 29, 2018.

Our systematic literature search in PubMed and Web of Knowledge was performed from inception of the databases to March 29, 2018, and it yielded 2,039 hits. Exclusion and inclusion criteria are shown in Fig 1. We removed 1,957 articles after screening titles and abstracts. After reading the manuscripts of the remaining articles, we excluded two cohort studies [15, 16] that did not assess physical activity on an individual level, two case-control studies [17, 18] that used cancer patients as controls, and 32 articles that were irrelevant. We included 1 cohort study of cardiorespiratory fitness and melanoma [19], 3 cohort studies of physical activity and melanoma [20–22] and 5 case-control studies of physical activity and melanoma [23–27] in our systematic review. We did not identify any other relevant studies by searching the reference lists of relevant articles. One [20] of the 3 included cohort studies of physical activity and melanoma combined information from 12 individual cohorts in a pooled analysis. For the main analysis, we used the pooled risk estimate from that study [20] because it was adjusted for adiposity. In sensitivity analyses and in sub-analyses stratified by gender and geographic region, we used adiposity-unadjusted estimates from that study [20] because published adiposity-adjusted estimates were not available.

Because there was only one investigation of cardiorespiratory fitness and melanoma [19], we were unable to perform a meta-analysis of cardiorespiratory fitness and melanoma risk. We did not combine studies of physical activity and cardiorespiratory fitness because physical activity and cardiorespiratory fitness represent independent risk factors for chronic diseases [28–30].

### Extraction of data

Two researchers (GB, TN) conducted independent data extractions from the identified studies. Any disagreement was resolved by discussion. We included relative risks (RR) with 95% confidence intervals (CI) for all available physical activity domains and for any timing in life of physical activity. We used the most comprehensively adjusted risk estimates provided that those risk estimates were also adjusted for age and, if applicable, gender. If available, we extracted separate risk estimates for men and women. In a sensitivity analysis, we included only one risk estimate per study and gender. For that sensitivity analysis, we preferred recreational physical activity and recent past physical activity over all other domains and timings in life of physical activity.

### Classification of timing in life of physical activity

We classified levels of physical activity as recent past physical activity if they referred to a time period less than 10 years prior to the baseline assessment (cohort studies) or cancer diagnosis (case-control studies). If those physical activity levels referred to a period further back in time, the physical activity variable was classified as distant past physical activity. An exception was made if that variable captured more than one time period in the past and if the utmost time periods were ≥10 years apart. In that case, the physical activity variable was classified as a measure of consistent physical activity over time. We considered occupational physical activity to fall into the latter category because people usually hold occupations for more than one decade.

### Study quality assessment and adjustments for UV radiation-related risk factors

We used the Monninkhof score [31] that was specifically developed for studies of physical activity and cancer incidence to assess the methodologic quality of the included studies, in particular the potential for selection bias (N = 5 items), misclassification (N = 11 items), and confounding (N = 3 items), which were weighted using a proportion of 2:2:1 with a maximum attainable score of 105 points. In one item (9 points), we chose adjustment for sun exposure and sun sensitivity as proxies for UV radiation-related skin damage [32, 33] on an individual level to assess the degree of control for major potential confounding factors. Studies adjusting for sun exposure on an individual level considered sun exposure during holidays 20 years prior to the interview [24], total lifetime sun exposure [27], recreational lifetime sun exposure [25] and occupational lifetime sun exposure [25]. Studies adjusting for sun sensitivity used one or more of the following variables: skin type [23–25], hair color [23, 25, 27], eye color [23], and immediate skin reaction to <30 minutes of UV radiation exposure at the beginning of the outdoor season [24]. One case-control study [24] additionally adjusted for UV radiation-related skin damage by including history of sunburns in childhood, actinic cheilitis, actinic keratosis, and solar lentigo in the model.

### Statistical methods

We interpreted all hazard ratios, incidence rate ratios, and odds ratios as relative risks. We summarized melanoma risk estimates comparing the highest and lowest physical activity categories on the log-scale in precision-weighted random effects meta-regression models [34] using the restricted maximum likelihood method (REML). We used funnel plots, Begg’s and Egger’s tests, and the Q- and I^2^-statistics to assess potential publication bias and between-study heterogeneity [35–37]. All statistical tests were two-sided and they were based on a significance level of 5%. All analyses were conducted in R, version 3.3.2, using the metafor-package [38, 39].

### Main analysis, stratified analyses, and sensitivity analyses

In the main analysis, we combined all available risk estimates of the association between physical activity and melanoma incidence, separately for cohort and case-control studies. In stratified analyses, we investigated the influence of the study quality score, gender, study geographic region, physical activity domain, timing in life of physical activity, physical activity measure, adjustments for UV radiation-related sun damage, sun sensitivity, sun exposure (on an individual level), smoking, adiposity, alcohol intake, and history of type 2 diabetes. In addition, we ran analyses stratified by adjustment for both sun sensitivity and sun exposure. To examine if any of the stratification variables explained the difference by study design, we repeated the stratified analyses for cohort and case-control studies combined. We compared summary estimates using likelihood ratio tests. In a sensitivity analysis, we included only one risk estimate per study and gender in the meta-analysis.

## Results

### Study characteristics

We considered 3 cohort studies [20–22] (*N* = 12,605 cases, of which the pooled study [20] contributed 12,438 cases, Table 1) and 5 case-control studies [23–27] (*N* = 1,295 cases, Table 1) of physical activity and melanoma risk, and 1 cohort study [19] (*N* = 49 cases, Table 2) of cardiorespiratory fitness and melanoma risk.

**Table 1:** Characteristics of the 3 cohort studies (*N* = 12,605 cases) and 5 case-control studies (*N* = 1,295 cases) on physical activity and melanoma risk included in the systematic review and meta-analysis grouped by study design.

Authors, year, study name, study country, study geographic region	Gender	Cases (N)	Physical activity domain, timing in life, measure	Relative risk (95% confidence interval), high vs. low physical activity	Low physical activity defined by	High physical activity defined by	Adjustment and matching factors (excluding age, sex)	Study quality score (%)
**Cohort studies**								
Moore et al., 2016^a^ [20]								
pooled analysis of 12 cohorts	men and women combined	12,438	recreational, recent past, energy expenditure	1.28 (1.17, 1.41)	bottom decile of energy expenditure	top decile of energy expenditure	adiposity (BMI), alcohol intake, education, race/ethnicity, smoking	62
Breast Cancer Detection and Demonstration Project (USA, North America)	women	278	recreational, recent past, energy expenditure	1.37 (0.98, 1.92)	bottom decile of energy expenditure	top decile of energy expenditure	alcohol intake, education, race/ethnicity, smoking	
Cancer Prevention Study II (USA, North America)	men and women combined	1,999	recreational, recent past, energy expenditure	1.28 (1.13, 1.45)	see above	see above	see above	
Cohort of Swedish Men (Sweden, Europe)	men	201	recreational, recent past, energy expenditure	1.90 (1.25, 2.88)	see above	see above	see above	
European Prospective Investigation into Cancer and Nutrition (several countries in Europe)	men and women combined	2,768	recreational, recent past, energy expenditure	1.47 (1.31, 1.64)	see above	see above	see above	
Iowa Women's Health Study (USA, North America)	women	283	recreational, recent past, frequency	1.10 (0.79, 1.54)	bottom decile of frequency of physical activity	top decile of frequency of physical activity	see above	
National Institutes of Health—AARP Diet and Health Study (USA, North America)	men and women combined	5,305	recreational, recent past, energy expenditure	1.23 (1.13, 1.33)	bottom decile of energy expenditure	top decile of energy expenditure	see above	
Physicians' Health Study I and II (USA, North America)	men	446	recreational, recent past, energy expenditure	1.20 (0.91, 1.57)	see above	see above	see above	
Prostate, Lung, Colorectal and Ovarian Cancer Screening Trial (USA, North America)	men and women combined	422	recreational, recent past, energy expenditure	0.99 (0.75, 1.31)	see above	see above	see above	
Swedish Mammography Cohort (Sweden, Europe)	women	106	recreational, recent past, energy expenditure	1.45 (0.83, 2.53)	see above	see above	see above	
US Radiologic Technologists Cohort (USA, North America)	men and women combined	307	recreational, recent past, energy expenditure	0.96 (0.70, 1.32)	see above	see above	see above	
Women's Health Study (USA, North America)	women	258	recreational, recent past, energy expenditure	1.71 (1.20, 2.42)	see above	see above	see above	
Women's Lifestyle and Health Study (Sweden, Europe)	women	65	recreational, recent past, energy expenditure	0.81 (0.41, 1.61)	see above	see above	see above	
Paffenbarger et al., 1987 [21]								
College Alumni Health Study (USA, North America)	men and women combined	59	recreational, distant past, duration	1.05 (0.57, 1.94)	less than 5 hours of vigorous physical activity per week	5 hours of vigorous physical activity per week or more	birth year	61
Veierod et al., 1997 [22]								
Norwegian National Health Screening Service (Norway, Europe)	men and women combined	108	recreational, recent past, qualitative	1.60 (0.40, 7.00)	sedentary	regular hard physical training	socio-economic status/sun exposure (ecologic level: geographic region)	60
			occupational, consistent, qualitative	1.20 (0.70, 2.30)	sedentary	heavy manual work	see above	64
**Case-control studies**								
Gogas et al., 2008 [23]								
Case-control study in Athens (Greece, Europe)	men and women combined	55	recreational, recent past, duration	0.70 (0.51, 0.94)		continuous variable for duration of physical exercise (increments in 30 min per day)	adiposity (BMI, waist-hip-ratio), alcohol intake, diet (plant food, animal food), education, serum leptin level, smoking, socio-economic status/sun exposure (ecologic level: residential area), sun sensitivity (skin type, hair color, eye color), type 2 diabetes	71
Kaskel et al., 2001 [24]								
Case-control study in Munich (Germany, Europe)	men and women combined	166	recreational, distant past, qualitative	0.30 (0.10, 1.10)	engaged rarely or never in at least one of the following outdoor activities in childhood: walking, playing soccer, cycling, athletics or gardening	engaged sometimes or regularly in at least one of the following outdoor activities in childhood: walking, playing soccer, cycling, athletics or gardening	family history of melanoma, sun exposure (individual level: sun exposure during holidays 20 years prior to the interview), sun sensitivity (skin type, immediate skin reaction to <30 minutes of UV radiation exposure at the beginning of the outdoor season), socio-economic status (ecologic level: residential area), UV radiation-related skin damage (sunburns in childhood, actinic cheilitis, actinic keratosis, solar lentigo)	57
Lee et al., 2009 [25]								
Western Canada Melanoma Study (Canada, North America)	men	231	occupational, consistent, energy expenditure	1.55 (0.76, 3.15)	bottom quintile	top quintile	socio-economic status (ecologic level: geographic region), sun exposure (individual level: lifetime occupational sun exposure, lifetime recreational sun exposure), sun sensitivity (hair color, skin color)	61
	women	355	occupational, consistent, energy expenditure	1.50 (0.77, 2.92)	see above	see above	see above	61
Parent et al., 2011 [26]								
Case-control study in Montreal (Canada, North America)	men	103	occupational, consistent, qualitative	0.14 (0.03, 0.70)	spent 75% of work years or more in sedentary jobs	spent 75% of work years or more in very active jobs	adiposity (BMI), diet (beta-carotene intake), education, physical activity (recreational), race/ethnicity, respondent status, smoking, socio-economic status (individual level: family income), sun exposure (ecologic level: residential area)	58
			recreational, consistent, frequency	1.24 (0.73, 2.12)	else	engaged in sports or outdoor activities regularly at least once per week for the duration of 6 months or more during adult life	adiposity (BMI), diet (beta-carotene intake), education, physical activity (occupational), race/ethnicity, respondent status, smoking, socio-economic status (individual level: family income), sun exposure (ecologic level: residential area)	71
			total, consistent, qualitative	0.86 (0.51, 1.42)	else	high occupational physical activity with or without regular recreational physical activity; alternatively, intermediate occupational physical activity combined with regular recreational physical activity	adiposity (BMI), diet (beta-carotene intake), education, race/ethnicity, respondent status, smoking, socio-economic status (individual level: family income), sun exposure (ecologic level: residential area)	62
Shors et al., 2001 [27]								
Case-control study in Washington State (USA, North America)	men	200	recreational, recent past, frequency	0.68 (0.36, 1.30)	no regular physical exercise	5–7 days per week	diet (fruit and vegetable intake), socio-economic status (ecologic level: geographic region), sun exposure (individual level: total lifetime sun exposure), sun sensitivity (hair color)	52
	women	185	recreational, recent past, frequency	0.65 (0.36, 1.70)	see above	see above	see above	52

BMI = body mass index; UV = ultraviolet.

^a^ Footnote to Moore et al. 2016 [20]: We used the pooled risk estimate reported in Moore et al. 2016 in the main analysis and in all stratified analyses with two exceptions: we used the sub-cohort-specific risk estimates reported in Moore et al. 2016 for stratification by gender and geographic region because sub-cohorts varied by gender and geographic region. In contrast to the pooled risk estimate, which was used for all other analyses, the sub-cohort-specific risk estimates were not adjusted for body mass index (BMI). However, Moore et al. 2016 reported that adjustment for BMI did not materially alter the risk estimates.

**Table 2:** Characteristics of the one cohort study (N = 49 cases) on cardiorespiratory fitness and melanoma risk included in the systematic review.

Authors, year, study name, study country, study geographic region	Gender	Cases (N)	Timing in life of cardio-respiratory fitness, measure	Relative risk (95% confidence interval), high vs. low cardiorespiratory fitness	Low cardio-respiratory fitness defined by	High cardio-respiratory fitness defined by	Adjustment and matching factors (excluding age, sex)	Study quality score (%)
**Cohort study**								
Robsahm et al., 2017 [19]								
Oslo Ischemia Study (Norway, Europe)	men	49	recent past, incremental bicycle exercise test	2.19 (0.99, 4.96)	bottom tertile (corresponding to <118 kJ/kg)	top tertile (corresponding to >161 kJ/kg)	adiposity (BMI), smoking	59

BMI = body mass index.

The one melanoma risk estimate [19] for cardiorespiratory fitness was based on middle-aged men residing in Norway and it was positive but not statistically significant (RR = 2.19, 95% CI = 0.99–4.96). It was adjusted for smoking and adiposity but not for UV-radiation related risk factors.

Cohort and case-control studies of physical activity and melanoma risk differed with respect to their gender distribution. Specifically, case-control studies reported separate risk estimates for men [25–27] and women [25, 27], whereas most cohorts examined melanoma risk in women [20] or in men and women combined [20–22]. Cohort and case-control studies had in common that there were as many studies from Europe [20, 22–24] as there were from North America [20, 21, 25–27].

Most cohort [20–22] and case-control studies [23, 24, 26, 27] of physical activity and melanoma risk examined recreational physical activity. Studies used various physical activity assessments including physical activity frequency [26, 27], physical activity duration [21, 23], energy expenditure [20, 25], and qualitative ratings [22, 24, 26].

None of the included cohort studies and four [23–25, 27] of the five included case-control studies of physical activity and melanoma risk adjusted for sun sensitivity or sun exposure on an individual level. However, two cohort studies used geographic region [22] and ground-level solar UV radiation [20] (erythemal dose; for a sub-cohort) to assess sun exposure on an ecologic level. The latter study [20] observed a statistically significant positive association between physical activity and melanoma incidence in geographic areas of high UV exposure and a statistically non-significant positive association between physical activity and melanoma incidence in geographic areas of low UV exposure. The former study [22] was only conducted in geographic areas of low UV exposure and it also observed a statistically non-significant positive association between physical activity and melanoma incidence.

One third of the cohort and case-control studies of physical activity and melanoma risk adjusted for smoking and adiposity [20, 23, 26], and one case-control study adjusted for type 2 diabetes [23].

### Main analysis

**Fig 2:**
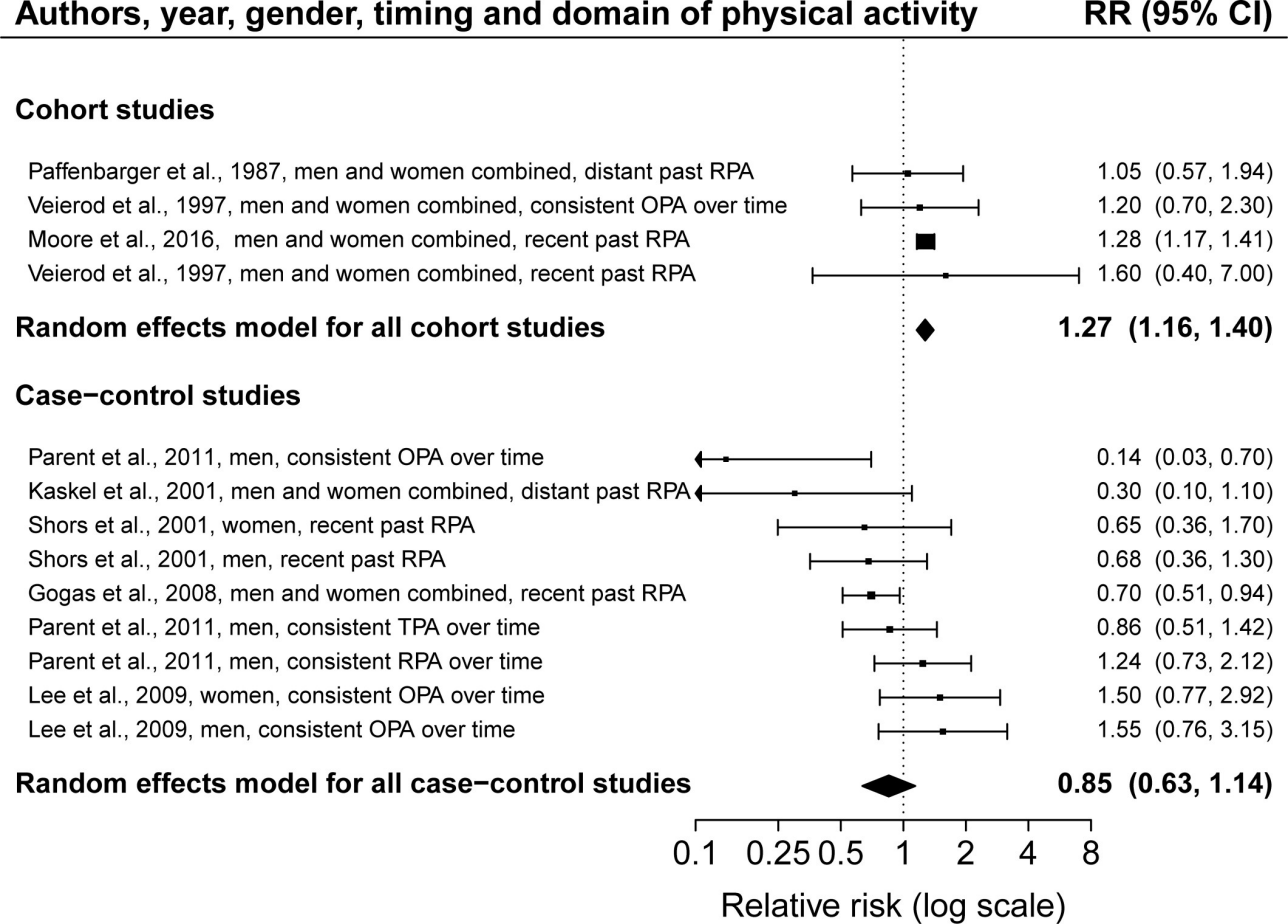
Forest plot of a random effects meta-analysis including 13 risk estimates of melanoma for a high versus low level of physical activity, grouped by study design; among cohorts: I^2^ = 0%, P-heterogeneity = 0.92; among case-control studies: I^2^ = 43%, P-heterogeneity = 0.03; P-difference by study design = 0.02. Abbreviations: RR, relative risk; CI, confidence interval; RPA, recreational physical activity, OPA, occupational physical activity; TPA, total physical activity.

**Fig 3:**
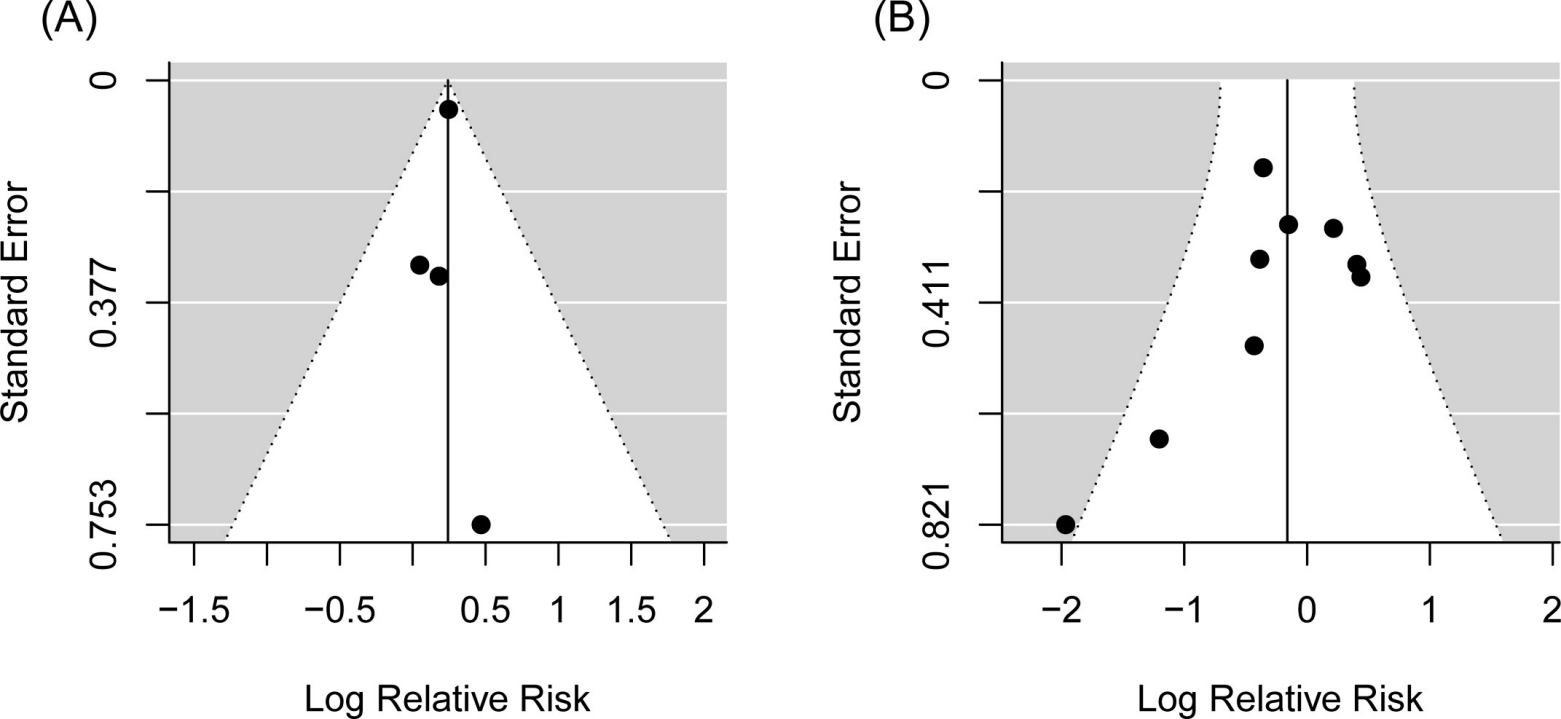
A) Funnel plot for random effects meta-analysis including 4 risk estimates of melanoma for a high versus low level of physical activity among cohort studies: P-value for Begg’s test = 0.99; P-value for Egger’s test = 0.79. B) Funnel plot for random effects meta-analysis including 9 risk estimates of melanoma for a high versus low level of physical activity among case-control studies: P-value for Begg’s test = 0.48; P-value for Egger’s test = 0.07.

We observed a statistically significant difference between the summary melanoma risk estimates for physical activity from cohort studies and case-control studies (*P*-difference = 0.02, Fig 2). Cohort studies indicated a statistically significant risk increase of 27% for melanoma when comparing physically active participants with physically inactive participants (RR = 1.27, 95% CI = 1.16–1.40), while case-control studies showed a statistically non-significant risk reduction of 15% for melanoma for the same comparison (RR = 0.85, 95% CI = 0.63–1.14). We found no indication of publication bias among cohort and case-control studies (cohort studies: *P*-value for Begg’s test = 0.99; *P*-value for Egger’s test = 0.79, Fig 3A; case-control studies: *P*-value for Begg’s test = 0.48; *P*-value for Egger’s test = 0.07, Fig 3B). We observed between-study heterogeneity among case-control studies but not among cohort studies (cohort studies: I^2^ = 0%, *P*-heterogeneity = 0.92; case-control studies: I^2^ = 43%, *P*-heterogeneity = 0.03).

### Stratified analyses and sensitivity analyses

We observed a stronger positive association between physical activity and melanoma in studies from Europe than studies from North America among cohort studies but not among case-control studies (Table 3). In contrast, study quality and timing in life of physical activity influenced the risk estimates for physical activity and melanoma risk among case-control studies but not among cohort studies. Case-control studies with a higher study quality score reported a null association between physical activity and melanoma risk. In contrast, case-control studies with a lower study quality score revealed a statistically significant inverse association between the two.

**Table 3:** Random effects summary estimates of melanoma risk for a high versus low level of physical activity by selected participant and design characteristics, evaluated separately for cohort and case-control studies and, in sensitivity analyses, for cohort and case-control studies combined.

	Cohort studies			Case-control studies			All cohort and case-control studies combined		
Stratification criterion	Number of included studies (number of included estimates)	RR^a^ (95% CI)	*P*-difference^b^	Number of included studies (number of included estimates)	RR^a^ (95% CI)	*P*-difference^b^	Number of included studies (number of included estimates)	RR^a^ (95% CI)	*P*-difference^b^
Total melanoma risk	3 (4)	1.27 (1.16, 1.40)		5 (9)	0.85 (0.63, 1.14)		8 (13)	0.98 (0.79, 1.22)	
Study design									
Cohort studies	3 (4)	1.27 (1.16, 1.40)		—	—		3 (4)	1.27 (1.16, 1.40)	
Case-control studies	—	—	—	5 (9)	0.85 (0.63, 1.14)	—	5 (9)	0.85 (0.63, 1.14)	0.02
Study quality score^c^									
Greater than or equal to the median	2 (2)	1.28 (1.16, 1.41)		3 (5)	1.03 (0.74, 1.44)		5 (7)	1.10 (0.86, 1.41)	
Less than the median	2 (2)	1.12 (0.63, 1.97)	0.65	3 (4)	0.50 (0.30, 0.86)	0.03	5 (6)	0.68 (0.43, 1.06)	0.06
Gender^d^									
Men	1 (2)	1.47 (0.94, 2.30)		3 (5)	0.91 (0.61, 1.36)		4 (7)	1.11 (0.80, 1.53)	
Women	1 (5)	1.32 (1.07, 1.62)	0.65^e^	2 (2)	1.06 (0.48, 2.38)	0.68^e^	3 (7)	1.29 (1.07, 1.57)	0.56^e^
Men and women combined	3 (8)	1.23 (1.09, 1.38)		2 (2)	0.58 (0.30, 1.15)		5 (10)	1.10 (0.92, 1.31)	
Study geographic region^d^									
Europe	2 (6)	1.47 (1.32, 1.63)		2 (2)	0.58 (0.30, 1.15)		4 (8)	1.13 (0.81, 1.58)	
North America	2 (9)	1.23 (1.15, 1.30)	0.004	3 (7)	0.97 (0.71, 1.33)	0.16	5 (16)	1.21 (1.14, 1.29)	0.84
Physical activity domain									
Recreational	3 (3)	1.28 (1.16, 1.40)		4 (5)	0.76 (0.55, 1.03)		7 (8)	0.93 (0.70, 1.23)	
Occupational	1 (1)	1.20 (0.63, 2.30)	0.86^f^	2 (3)	0.82 (0.21, 3.19)	0.23^f^	3 (4)	1.06 (0.55, 2.06)	0.41^f^
Total	—	—		1 (1)	0.86 (0.51, 1.45)		1 (1)	0.86 (0.51, 1.45)	
Timing in life of physical activity									
Recent past	2 (2)	1.28 (1.16, 1.41)		2 (3)	0.69 (0.53, 0.91)		4 (5)	0.90 (0.62, 1.31)	
Distant past	1 (1)	1.05 (0.57, 1.94)		1 (1)	0.30 (0.08, 1.10)		2 (2)	0.64 (0.19, 2.13)	
Consistent over time	1 (1)	1.20 (0.63, 2.30)	0.81	2 (5)	1.10 (0.80, 1.52)	0.02	3 (6)	1.12 (0.86, 1.47)	0.59
Physical activity measure									
Frequency	—	—		2 (3)	0.88 (0.56, 1.41)		2 (3)	0.88 (0.56, 1.41)	
Duration	1 (1)	1.05 (0.57, 1.94)		1 (1)	0.70 (0.51, 0.96)		2 (2)	0.78 (0.55, 1.12)	
Energy expenditure	1 (1)	1.28 (1.16, 1.41)		1 (2)	1.52 (0.94, 2.48)		2 (3)	1.29 (1.17, 1.42)	
Qualitative ratings	1 (2)	1.26 (0.69, 2.28)	0.82	2 (3)	0.41 (0.14, 1.20)	0.16	3 (5)	0.69 (0.35, 1.39)	0.001
Adjustment for UV radiation-related skin damage^g^									
Yes	—	—		1 (1)	0.30 (0.08, 1.10)		1 (1)	0.30 (0.08, 1.10)	
No	3 (4)	1.27 (1.16, 1.40)	—	4 (8)	0.89 (0.67, 1.18)	0.13	7 (12)	1.01 (0.82, 1.25)	0.09
Adjustment for sun sensitivity^h^									
Yes	—	—		4 (6)	0.85 (0.58, 1.24)		4 (6)	0.85 (0.58, 1.24)	
No	3 (4)	1.27 (1.16, 1.40)	—	1 (3)	0.68 (0.24, 1.89)	0.93	4 (7)	1.17 (0.98, 1.40)	0.13
Adjustment for sun exposure on an individual level^i^									
Yes	—	—		3 (5)	0.90 (0.54, 1.50)		3 (5)	0.90 (0.54, 1.50)	
No	3 (4)	1.27 (1.16, 1.40)	—	2 (4)	0.80 (0.55, 1.15)	0.64	5 (8)	1.00 (0.77, 1.28)	0.80
Adjustment for sun sensitivity^h^ and sun exposure on an individual level^i^									
Adjusted for sun sensitivity and sun exposure on an individual level	—	—		3 (5)	0.90 (0.54, 1.50)		3 (5)	0.90 (0.54, 1.50)	
Adjusted for sun sensitivity but not for sun exposure on an individual level	—	—		1 (1)	0.70 (0.51, 0.96)		1 (1)	0.70 (0.51, 0.96)	
Adjusted for sun exposure on an individual level but not for sun sensitivity	—	—		—	—		—	—	
Not adjusted for sun sensitivity and sun exposure on an individual level	3 (4)	1.27 (1.16, 1.40)	—	1 (3)	0.68 (0.24, 1.89)	0.91	4 (7)	1.17 (0.98, 1.40)	0.15
Adjustment for adiposity									
Yes	1 (1)	1.28 (1.16, 1.41)		2 (4)	0.80 (0.55, 1.15)		3 (5)	0.91 (0.63, 1.32)	
No	2 (3)	1.15 (0.75, 1.77)	0.64	3 (5)	0.90 (0.54, 1.50)	0.64	5 (8)	1.03 (0.77, 1.37)	0.73
Adjustment for type 2 diabetes									
Yes	—	—		1 (1)	0.70 (0.51, 0.96)		1 (1)	0.70 (0.51, 0.96)	
No	3 (4)	1.27 (1.16, 1.40)	—	4 (8)	0.88 (0.61, 1.26)	0.58	7 (12)	1.07 (0.88, 1.31)	0.10
Adjustment for smoking									
Yes	1 (1)	1.28 (1.16, 1.41)		2 (4)	0.80 (0.55, 1.15)		3 (5)	0.91 (0.63, 1.32)	
No	2 (3)	1.15 (0.75, 1.77)	0.64	3 (5)	0.90 (0.54, 1.50)	0.64	5 (8)	1.03 (0.77, 1.37)	0.73
Adjustment for alcohol intake									
Yes	1 (1)	1.28 (1.16, 1.41)		1 (1)	0.70 (0.51, 0.96)		2 (2)	0.97 (0.53, 1.74)	
No	2 (3)	1.15 (0.75, 1.77)	0.64	4 (8)	0.88 (0.61, 1.26)	0.58	6 (11)	0.99 (0.80, 1.24)	0.93

RR = relative risk; CI = confidence interval

^a^ RR comparing highest versus lowest physical activity level.

^b^ The P-difference values were obtained using meta-regression comparing the model including the stratification variable as explanatory variable with the null model not including any explanatory variables.

^c^ Among cohort studies, the quality score ranged from 61 to 64 percentage points (out of 100 percentage points), with a median cut-off at 62 percentage points. Among case-control studies, the quality score ranged from 52 to 71 percentage points (out of 100 percentage points), with a median cut-off at 61 percentage points. Among all studies, the quality score ranged from 52 to 71 percentage points (out of 100 percentage points), with a median cut-off at 61 percentage points.

^d^ For the analyses stratified by gender and geographic region, we used the sub-cohort-specific risk estimates from Moore et al. 2016 [20] because sub-cohorts varied by gender and geographic region. In contrast to the pooled risk estimate from Moore et al. 2016, which was used for all other analyses, the sub-cohort-specific risk estimates were not adjusted for body mass index (BMI). However, Moore et al. 2016 reported that adjustment for BMI did not materially alter the risk estimates.

^e^ Comparing risk estimates of men with risk estimates of women and disregarding risk estimates of men and women combined.

^f^ Comparing risk estimates of recreational activity with risk estimates of occupational activity and disregarding risk estimates of total activity.

^g^ Studies adjusting for UV radiation-related skin damage adjusted for at least one of the following risk factors: sunburns in childhood, actinic cheilitis, actinic keratosis, solar lentigo.

^h^ Studies adjusting for sun sensitivity adjusted for at least one of the following risk factors: skin type, hair color, eye color, immediate skin reaction to <30 minutes of UV radiation exposure at the beginning of the outdoor season.

^i^ Studies adjusting for sun exposure on an individual level adjusted for at least one of the following risk factors: sun exposure during holidays 20 years prior to the interview, total lifetime sun exposure, recreational lifetime sun exposure and occupational lifetime sun exposure.

Because case-control studies of lifetime physical activity and melanoma risk scored higher in the methodologic quality assessment than did case-control studies of recent or distant past physical activity, adjustment for timing in life of physical activity removed the statistically significant influence of study quality (*P*-difference across quality score before and after adjustment for timing in life of physical activity among case-control studies = 0.03 and 0.30, respectively). Similarly, the between-study heterogeneity among case-control studies was no longer apparent after adjusting for timing in life of physical activity (I^2^ = 0%; *P*-heterogeneity = 0.17). Case-control studies of recent and distant past physical activity observed statistically significant and non-significant inverse relations between physical activity and melanoma, respectively, whereas case-control studies of lifetime physical activity revealed a statistically non-significant positive association between the two (*P*-difference = 0.02, Table 3). In contrast, cohort studies found a statistically significant positive relation between recent past physical activity and melanoma risk and no statistically significant variation in risk estimates across timing in life of physical activity (Table 3).

When we repeated the stratified analyses among cohort and case-control studies combined in a sensitivity analysis, we found that the summary risk estimate that was based on energy expenditure as a measure of physical activity was positive, whereas summary risk estimates that were based on physical activity frequency, physical activity duration, or qualitative ratings were inverse (*P*-difference = 0.001, Table 3). The energy expenditure-based risk estimate was driven by the pooled cohort study [20].

In an additional sensitivity analysis, we allowed only one risk estimate per study and gender, which yielded comparable summary risk estimates to those from the main analysis (RR among cohort studies = 1.28, 95% CI = 1.16–1.40; RR among case-control studies = 0.91, 95% CI = 0.65–1.26). Similarly, using all 12 individual risk estimates rather than the pooled risk estimate from the pooled cohort study [20] did not materially alter the results (S1 Table, S1 Fig).

## Discussion

The primary new finding of the current meta-analysis is that the association between physical activity and melanoma differs by study design. Specifically, we found a statistically significant positive association between physical activity and melanoma incidence among cohort studies, whereas we observed a statistically non-significant inverse relation among case-control studies. The one cohort study of cardiorespiratory fitness and melanoma reported a positive but statistically non-significant association between the two.

The most important risk factor for melanoma is UV radiation-related skin damage, which includes a history of sunburns, dysplastic and common naevi, actinic keratosis, and solar lentigo [40–43]. Additional melanoma risk factors are those that enhance susceptibility to UV radiation-related skin damage, such as skin phototype, hair color, eye color, freckles, the ability to tan, and the skin’s immediate reaction to sun exposure at the beginning of the tanning season [42].

The presence of melanoma at sites that are rarely exposed to UV radiation and the relations of naevus patterns (site and count) to the histology and location of melanoma suggest that there are diverging pathways of melanoma incidence [44–46]. One recent study [47] reported that the observed positive relation of bone mineral density to naevus count (and thereby to melanoma incidence) could be partly explained by leucocyte telomere length. However, it remains unclear whether that association is causal because that study did not adjust for sun exposure as a potential confounding factor and because leucocyte telomere length has been inversely related to sun exposure, naevus count, and melanoma incidence [47, 48]. Similarly, it is uncertain whether physical activity affects leucocyte telomere length and naevus count [49–51]. A large cross-sectional study reported a positive association between physical activity and naevus count among 26,000 men but not among 67,000 women in age-adjusted analyses [51]. That observed gender difference could be due to confounding by sun exposure because women are less likely to participate in outdoor physical activities than men and if they do, they have lower vitamin D serum levels than men in each outdoor physical activity category [52, 53]. Carefully designed future studies are required to examine if physical activity affects leucocyte telomere length, naevus count, and melanoma incidence independently of sun exposure.

Moreover, various genetic factors have been related to the incidence of melanoma, including genes related to pigmentation and melanin production, the development of naevi, DNA repair, and family history of melanoma [8, 54, 55]. Immunosuppressive therapy or immunosuppressive UV radiation-related cell damage also increase the risk of melanoma [7]. In addition, adiposity, chronic inflammation, and oxidative stress have all been positively related to melanoma incidence [6, 8, 9, 56].

Mechanistically, physical activity and cardiorespiratory fitness may prevent melanoma through enhanced immune function, increased DNA repair capacity, reduced oxidative stress, decreased chronic inflammation, and weight control [10–13, 57–61]. Thus, a potential preventive role of physical activity and cardiorespiratory fitness for the development of melanoma is biologically plausible. However, it may be challenging for epidemiologic studies to detect a potential protective effect of physical activity and cardiorespiratory fitness without comprehensive adjustment for UV radiation-related skin damage. In fact, incomplete adjustment for UV radiation-related skin damage may residually confound the relations of physical activity and cardiorespiratory fitness to melanoma because physical activity and cardiorespiratory fitness are positively associated with time spent outdoors, sunburns, and UV radiation-related skin damage [62–65].

Because none of the three cohort studies of physical activity and melanoma included in the present meta-analysis were adjusted for UV radiation-related skin damage, sun sensitivity, or sun exposure (on an individual level), the statistically significant positive summary risk estimate for physical activity and melanoma risk obtained from cohort studies may be a result of major confounding by UV radiation-related risk factors. The greater magnitude of the risk estimate among studies from Europe as compared to that from studies from North America indicates a greater degree of confounding of the physical activity and melanoma relation in European than North American populations, potentially due to lower levels of skin pigmentation [66] and greater levels of outdoor physical activity [67] among Europeans than North Americans. In line with this, one pooled prospective analysis [20] reported that the positive association between physical activity and melanoma was more pronounced in geographic areas of high UV radiation exposure than in areas of low UV radiation exposure. Interestingly, one case-control study [24] that was able to adjust for UV radiation-related skin damage observed a strong but statistically non-significant inverse association between melanoma and physical activity in childhood, a time period during which sunburns pose one of the strongest risk factors for the development of subsequent melanoma [68].

Four of the five included case-control studies attempted to minimize the potential for UV radiation-related confounding by detailed adjustment for UV radiation-related skin damage, sun sensitivity (skin phototype, hair color, eye color, and immediate skin reaction to UV radiation), and sun exposure (on an individual level). Thus, it is conceivable that the statistically non-significant inverse summary risk estimate for physical activity and melanoma risk obtained from the included case-control studies is closer to the true association between physical activity and melanoma than that obtained from the cohort studies.

Because the included studies used a large variety of physical activity assessments and because there was no single predominant type of physical activity assessment, in our main analysis we summarized risk estimates across all types of physical activity assessments to obtain a summary risk estimate of melanoma incidence for an “average” physical activity assessment. As a consequence, we included more than one risk estimate per gender in our main analysis for one [22] of the three cohort studies and for one [26] of the five case-control studies. We therefore verified that results from the main analysis combining all types of physical activity assessments were comparable to those from a rigorous sensitivity analysis allowing only one risk estimate per study and gender. In addition, we stratified analyses by physical activity domain, timing in life of physical activity, and type of physical activity measure but we did not find any statistically significant influence of those variables on the association between physical activity and melanoma incidence with one exception: among case-control studies, the risk estimate for consistent physical activity over time tended to be positive, while those for recent past physical activity and distant past physical activity tended to be inverse. This observation may be explained by the potential of residual confounding through incomplete adjustment for UV radiation-related risk factors in some of the case-control studies of consistent physical activity over time.

The major strength of the present systematic review and meta-analysis of physical activity and melanoma risk is the large number of cases and the inclusion of both cohort and case-control studies. Considering both study types was essential because case-control studies were methodologically superior to cohort studies in their degree of adjustment for UV radiation-related risk factors. Although case-control studies are generally considered to be prone to recall and selection biases, case-control studies of melanoma risk factors have not been subject to notable recall bias [69–74]. Also, the included case-control studies attempted to reduce the potential for selection bias by choosing population-based controls [23, 25–27] or a wide variety of hospital-based controls [24] and by adjusting for variables predictive of participants’ response rates, including age, sex, education, and socio-economic status [23–27, 75–77]. We did not identify any publication bias. However, due to the limited number of studies available for meta-analysis, the power of the statistical tests for publication bias was low.

In summary, we observed significant heterogeneity between summary risk estimates from cohort and case-control studies of physical activity and melanoma incidence. It is likely that the statistically significant positive association between physical activity and melanoma risk and the positive but statistically not significant association between cardiorespiratory fitness and melanoma observed in cohort studies is the result of incomplete adjustment for UV radiation-related skin damage. Case-control studies of physical activity and melanoma generally adjusted for UV radiation-related risk factors and produced a statistically non-significant inverse relation between physical activity and melanoma incidence. To clarify the true nature of the relations of physical activity and cardiorespiratory fitness to melanoma incidence, future prospective studies are required to examine physical activity and cardiorespiratory fitness in relation to melanoma risk after careful adjustment for UV radiation-related skin damage.

## Supporting information

S1 File Literature search strategy. (DOCX)

S2 File PRISMA checklist. (DOC)

S1 Table Random effects summary estimates of melanoma risk for a high versus low level of physical activity by selected participant and design characteristics using the 12 individual risk estimates rather than the pooled risk estimate from Moore et al. 2016 [20] as sensitivity analysis to Table 3. (DOCX)

S1 Fig Funnel plot for random effects meta-analysis including 15 risk estimates of melanoma for a high versus low level of physical activity among cohort studies in the sensitivity analysis including the 12 individual risk estimates rather than the pooled risk estimate from Moore et al. 2016 [20] as sensitivity analysis to Fig 3B: P-value for Begg’s test = 0.77; P-value for Egger’s test = 0.70. (EPS)
