# Supplementary material for: Physical activity, cardiorespiratory fitness and risk of cutaneous malignant melanoma: Systematic review and meta-analysis
Source: PLoS One. 2018 Oct 31;13(10):e0206087. doi: 10.1371/journal.pone.0206087 (PMC6209223; doi:10.1371/journal.pone.0206087)
Supplement: S1 File — (DOCX) [file pone.0206087.s001.docx]

# Supplementary File

## Literature search strategy

The PubMed and Web of Knowledge search was last updated on March 29, 2018. The following terms were pasted, all at once, into the search command line:

(physical activity [title/abstract] OR exercis* [title/abstract] OR cardiorespiratory fitness [title/abstract] OR cardiovascular fitness [title/abstract] OR resistance training [title/abstract] OR endurance training [title/abstract] OR aerobic [title/abstract] OR sport [title/abstract] OR sports [title/abstract] OR athletes [title/abstract] OR players [title/abstract] OR outdoor activities [title/abstract] OR lifestyle [title/abstract])

AND (melanoma [title] OR skin cancer [title] OR cancer [title] OR cancers [title])

NOT (review [ptyp] OR meta-analysis [ptyp] OR editorial [ptyp] OR guideline [ptyp] OR news [ptyp])

NOT (lung* [title] NOT melanoma [title] NOT skin cancer [title])

NOT (bronchial [title] NOT melanoma [title] NOT skin cancer [title])

NOT (breast* [title] NOT melanoma [title] NOT skin cancer [title])

NOT (mamma* [title] NOT melanoma [title] NOT skin cancer [title])

NOT (ovar* [title] NOT melanoma [title] NOT skin cancer [title])

NOT (endometr* [title] NOT melanoma [title] NOT skin cancer [title])

NOT (uter* [title] NOT melanoma [title] NOT skin cancer [title])

NOT (cervi* [title] NOT melanoma [title] NOT skin cancer [title])

NOT (gynecolog* [title] NOT melanoma [title] NOT skin cancer [title])

NOT (prostat* [title] NOT melanoma [title] NOT skin cancer [title])

NOT (testic* [title] NOT melanoma [title] NOT skin cancer [title])

NOT (urinary* [title] NOT melanoma [title] NOT skin cancer [title])

NOT (bladder* [title] NOT melanoma [title] NOT skin cancer [title])

NOT (urothelial* [title] NOT melanoma [title] NOT skin cancer [title])

NOT (colon* [title] NOT melanoma [title] NOT skin cancer [title])

NOT (rectal* [title] NOT melanoma [title] NOT skin cancer [title])

NOT (colorectal* [title] NOT melanoma [title] NOT skin cancer [title])

NOT (bowel* [title] NOT melanoma [title] NOT skin cancer [title])

NOT (*digestive* [title] NOT melanoma [title] NOT skin cancer [title])

NOT (gastric* [title] NOT melanoma [title] NOT skin cancer [title])

NOT (stomach* [title] NOT melanoma [title] NOT skin cancer [title])

NOT (oesophag* [title] NOT melanoma [title] NOT skin cancer [title])

NOT (esophag* [title] NOT melanoma [title] NOT skin cancer [title])

NOT (pancrea* [title] NOT melanoma [title] NOT skin cancer [title])

NOT (tract* [title] NOT melanoma [title] NOT skin cancer [title])

NOT (duct* [title] NOT melanoma [title] NOT skin cancer [title])

NOT (tube* [title] NOT melanoma [title] NOT skin cancer [title])

NOT (liver* [title] NOT melanoma [title] NOT skin cancer [title])

NOT (hepatocellular [title] NOT melanoma [title] NOT skin cancer [title])

NOT (gallbladder* [title] NOT melanoma [title] NOT skin cancer [title])

NOT (oral* [title] NOT melanoma [title] NOT skin cancer [title])

NOT (pharyn* [title] NOT melanoma [title] NOT skin cancer [title])

NOT (nasopharyn* [title] NOT melanoma [title] NOT skin cancer [title])

NOT (oropharyn* [title] NOT melanoma [title] NOT skin cancer [title])

NOT (laryn* [title] NOT melanoma [title] NOT skin cancer [title])

NOT (lymphoid* [title] NOT melanoma [title] NOT skin cancer [title])

NOT (bone* [title] NOT melanoma [title] NOT skin cancer [title])

NOT (head and neck* [title] NOT melanoma [title] NOT skin cancer [title])

NOT (brain [title] NOT melanoma [title] NOT skin cancer [title])

NOT (meningioma* [title] NOT melanoma [title] NOT skin cancer [title])

NOT (glioma* [title] NOT melanoma [title] NOT skin cancer [title])

NOT (thora* [title] NOT melanoma [title] NOT skin cancer [title])

NOT (thyroid* [title] NOT melanoma [title] NOT skin cancer [title])

NOT (squamous cell* [title] NOT melanoma [title] NOT skin cancer [title])

NOT (basal cell* [title] NOT melanoma [title] NOT skin cancer [title])

NOT (adenoma* [title] NOT melanoma [title] NOT skin cancer [title])

NOT (multiple [title] NOT melanoma [title] NOT skin cancer [title]) NOT (mouth [title] NOT melanoma [title] NOT skin cancer [title])

NOT (surviv* [title] OR prognosis* [title] OR quality of life* [title] OR fatigue* [title] OR pallia* [title] OR cancer patient* [title] OR cancer care* [title] OR recurrence* [title] OR progression* [title] OR clinical outcome* [title] OR chemotherapy* [title] OR radiation* [title] OR radiotherapy* [title] OR therap* [title] OR rehabilitation* [title] OR recovery* [title] OR cancer diagnosis [title] OR cancer treatment [title] OR cancer surgery [title] OR with cancer [title])
