# Supplementary material for: Physical activity, cardiorespiratory fitness and risk of cutaneous malignant melanoma: Systematic review and meta-analysis
Source: PLoS One. 2018 Oct 31;13(10):e0206087. doi: 10.1371/journal.pone.0206087 (PMC6209223; doi:10.1371/journal.pone.0206087)
Supplement: S1 Table — (DOCX) [file pone.0206087.s003.docx]

**S1 Table. Random effects summary estimates of melanoma risk for a high versus low level of physical activity by selected participant and design characteristics using the 12 individual risk estimates rather than the pooled risk estimate from Moore et al. 2016 [**[**20**](#_ENREF_20)**] as sensitivity analysis to Table 3.**

|  | **Cohort studies** | | |
| --- | --- | --- | --- |
| **Stratification criterion** | **Number of included studies (number of included estimates)** | **RR^a^ (95% CI)** | *P***-difference^b^** |
| Total melanoma risk | 3 (15) | 1.27 (1.16, 1.39) |  |
| Study design |  |  |  |
| Cohort studies | 3 (15) | 1.27 (1.16, 1.39) |  |
| Case-control studies | -- | -- | -- |
| Study quality score^c^ |  |  |  |
| Greater than or equal to the median | 2 (13) | 1.27 (1.16, 1.40) |  |
| Less than the median | 2 (2) | 1.12 (0.63, 1.97) | 0.68 |
| Gender |  |  |  |
| Men | 1 (2) | 1.47 (0.94, 2.30) |  |
| Women | 1 (5) | 1.32 (1.07, 1.62) | 0.65^d^ |
| Men and women combined | 3 (8) | 1.23 (1.09, 1.38) |  |
| Study geographic region |  |  |  |
| Europe | 2 (6) | 1.47 (1.32, 1.63) |  |
| North America | 2 (9) | 1.23 (1.15, 1.30) | 0.004 |
| Physical activity domain |  |  |  |
| Recreational | 3 (14) | 1.27 (1.16, 1.39) |  |
| Occupational | 1 (1) | 1.20 (0.63, 2.30) | 0.87^e^ |
| Total | -- | -- |  |
| Timing in life of physical activity |  |  |  |
| Recent past | 2 (13) | 1.28 (1.16, 1.40) |  |
| Distant past | 1 (1) | 1.05 (0.57, 1.94) |  |
| Consistent over time | 1 (1) | 1.20 (0.63, 2.30) | 0.83 |
| Physical activity measure |  |  |  |
| Frequency | 1 (1) | 1.10 (0.79, 1.54) |  |
| Duration | 1 (1) | 1.05 (0.57, 1.94) |  |
| Energy expenditure | 1 (11) | 1.29 (1.16, 1.42) |  |
| Qualitative ratings | 1 (2) | 1.26 (0.69, 2.28) | 0.83 |
| Adjustment for UV radiation-related skin damage^f^ |  |  |  |
| Yes | -- | -- |  |
| No | 3 (15) | 1.27 (1.16, 1.39) | -- |
| Adjustment for sun sensitivity^g^ |  |  |  |
| Yes | -- | -- |  |
| No | 3 (15) | 1.27 (1.16, 1.39) | -- |
| Adjustment for sun exposure on an individual level^h^ |  |  |  |
| Yes | -- | -- |  |
| No | 3 (15) | 1.27 (1.16, 1.39) | -- |
| Adjustment for sun sensitivity^g^ and sun exposure on an individual level^h^ |  |  |  |
| Adjusted for sun sensitivity and sun exposure on an individual level | -- | -- |  |
| Adjusted for sun sensitivity but not for sun exposure on an individual level | -- | -- |  |
| Adjusted for sun exposure on an individual level but not for sun sensitivity | -- | -- |  |
| Not adjusted for sun sensitivity and sun exposure on an individual level | 3 (15) | 1.27 (1.16, 1.39) | -- |
| Adjustment for adiposity |  |  |  |
| Yes | -- | -- |  |
| No | 3 (15) | 1.27 (1.16, 1.39) | -- |
| Adjustment for type 2 diabetes |  |  |  |
| Yes | -- | -- |  |
| No | 3 (15) | 1.27 (1.16, 1.39) | -- |
| Adjustment for smoking |  |  |  |
| Yes | 1 (12) | 1.27 (1.16, 1.40) |  |
| No | 2 (3) | 1.15 (0.75, 1.77) | 0.67 |
| Adjustment for alcohol intake |  |  |  |
| Yes | 1 (12) | 1.27 (1.16, 1.40) |  |
| No | 2 (3) | 1.15 (0.75, 1.77) | 0.67 |

RR=relative risk; CI=confidence interval

^a^ RR comparing highest versus lowest physical activity level.

^b^ The p-difference values were obtained using meta-regression comparing the model including the stratification variable as explanatory variable with the null model not including any explanatory variables.

^c^ For this sensitivity analysis, we used the same cut-off of 62 percentage points as median quality score as in Table 3.

^d^ Comparing risk estimates of men with risk estimates of women and disregarding risk estimates of men and women combined.

^e^ Comparing risk estimates of recreational activity with risk estimates of occupational activity and disregarding risk estimates of total activity.

^f^ Studies adjusting for UV radiation-related skin damage adjusted for at least one of the following risk factors: sunburns in childhood, actinic cheilitis, actinic keratosis, solar lentigo.

^g^ Studies adjusting for sun sensitivity adjusted for at least one of the following risk factors: skin type, hair color, eye color, immediate skin reaction to <30 minutes of UV radiation exposure at the beginning of the outdoor season.

^h^ Studies adjusting for sun exposure on an individual level adjusted for at least one of the following risk factors: sun exposure during holidays 20 years prior to the interview, total lifetime sun exposure, recreational lifetime sun exposure and occupational lifetime sun exposure.
